# Epidemiological characteristics, clinical presentations, and prognoses of pediatric brain tumors: Experiences of national center for children’s health

**DOI:** 10.3389/fonc.2023.1067858

**Published:** 2023-01-27

**Authors:** Wei Yang, Yingjie Cai, Jiashu Chen, Ping Yang, Zesheng Ying, Yuting Liang, Miao Ling, Kaiyi Zhu, Hailang Sun, Yuanqi Ji, Xiaojiao Peng, Nan Zhang, Wenping Ma, Ming Ge

**Affiliations:** ^1^ Department of Neurosurgery, Beijing Children’s Hospital, Capital Medical University, National Center for Children’s Health, Beijing, China; ^2^ Department of Cardiology, Shanxi Bethune Hospital, Shanxi Academy of Medical Sciences, Tongji Shanxi Hospital, Third Hospital of Shanxi Medical University, Taiyuan, China; ^3^ Department of Pathology, Beijing Children’s Hospital, Capital Medical University, National Center for Children’s Health, Beijing, China

**Keywords:** children, brain tumor, epidemiology, clinical presentation, prognosis

## Abstract

**Background:**

We aimed to describe the epidemiological characteristics, clinical presentations, and prognoses in a national health center for children.

**Methods:**

From January 2015 to December 2020, 484 patients aged 0-16 years, who were diagnosed with brain tumors and received neurosurgery treatment, were enrolled in the study. Pathology was based on the World Health Organization 2021 nervous system tumor classification, and tumor behaviors were classified according to the International Classification of Diseases for Oncology, third edition.

**Results:**

Among the 484 patients with brain tumors, the median age at diagnosis was 4.62 [2.19, 8.17] years (benign tumors 4.07 [1.64, 7.13] vs. malignant tumors 5.36 [2.78, 8.84], p=0.008). The overall male-to-female ratio was 1.33:1(benign 1.09:1 vs. malignant 1.62:1, p=0.029). Nausea, vomiting, and headache were the most frequent initial symptoms. The three most frequent tumor types were embryonal tumors (ET, 22.8%), circumscribed astrocytic gliomas (20.0%), and pediatric-type diffuse gliomas (11.0%). The most common tumor locations were the cerebellum and fourth ventricle (38.67%), the sellar region (22.9%) and ventricles (10.6%). Males took up a higher proportion than females in choroid plexus tumors (63.6%), ET (61.1%), ependymal tumors (68.6%), and germ cell tumors (GCTs, 78.1%). Patients were followed for 1 to 82 months. The overall 5-year survival rate was 77.5%, with survival rates of 91.0% for benign tumors and 64.6% for malignant tumors.

**Conclusion:**

Brain tumors presented particularly sex-, age-, and regional-dependent epidemiological characteristics. Our results were consistent with previous reports and might reflect the real epidemiological status in China.

## Background

According to the Central Brain Tumor Registry of the United States (CBTRUS) report, brain tumors and other central nervous system (CNS) tumors are the most common solid tumor in the population aged 0-14 years. Malignant brain tumors are the most common cause of death in this group. It is known that brain tumors have specific site distribution and predilection age patterns. The meninges are the most common tumor site in all age groups, with meningiomas being the most common tumor histology in the 2021 CBTRUS report (1, 2). However, brain tumors in the pediatric population have different epidemiological characteristics than those in the adult population. According to the CBTRUS 2016 report, the most common brain tumor site was the cerebellum, and gliomas were the most common histological group, of which pilocytic astrocytoma accounted for the majority of brain tumors among 0- to 14-year-old children (3).

An annual CBTRUS report characterized pediatric brain tumors in the US, which also hinted at racial differences among brain tumors. However, demographic data are seldom reported in Chinese children due to the lack of a nationwide tumor registration system. The only available study is Zhou’s (4) report, which summarized pediatric epidemiological characteristics with a single data source: Beijing Tiantan Hospital. However, their data were based on the World Health Organization (WHO) 2000 and were incomplete due to a lack of prognostic data. Hence, we reviewed all patients with brain tumors who received surgical treatment in Beijing Children’s Hospital, National Center for Children’s Health from 2015 to 2020, aiming to validate and update the epidemiological characteristics of pediatric brain tumors.

## Materials and methods

### Data source

From January 2015 to December 2020, patients between 0 and 16 years who were diagnosed with brain tumors and underwent neurosurgery were enrolled in the study. Duplicated data generated by tumor recurrence or other treatments were deleted. Patients who were hospitalized but refused neurosurgery or data from patients without a pathological diagnosis were excluded. Demographic information and clinical information, including medical history, initial symptoms, pathology, WHO grade, tumor location, surgery date, surgery duration, ventriculoperitoneal shunt (V-P shunt), average length of hospital stay, and medical expenditures, were collected. Patients were followed up by telephone or scheduled outpatient visits. The follow-up items included adjuvant treatment programs, survival status, tumor relapse, and date of death. This work was approved by ethic committee board of Beijing Children’s Hospital (IRB ID # [2021]:-E-232-Y).

### Classification

Histological diagnosis was based on the 2021 WHO Classification of Tumors of the Central Nervous System (2021 WHO classification) and was divided into 12 subgroups (5). Tumor behavior was labeled according to the International Classification of Diseases for Oncology, third edition (ICD-O-3), with behavior codes of 3 for malignant tumors and 0 or 1 for nonmalignant tumors. Low-grade gliomas (LGGs) included all the gliomas of WHO 1 and 2, and high-grade gliomas (HGGs) included that of WHO 3 and 4. In addition, neuronal and mixed neuronal-glial tumors were not categorized into gliomas.

### Anatomical locations of tumors

Tumor location referred to the categories of the CBTRUS report and topography code in the ICD-O-3. To make it more practical, some details were revised. The fourth ventricle and cerebellum were merged into one group, namely, the cerebellum or the fourth ventricle group. The ventricles here are referred as the lateral ventricles and third ventricle. The sellar region was used to replace the pituitary gland and craniopharyngeal duct, which included tumors from the pituitary gland and optic chiasma. Cranial nerves referred to all the cranial nerves apart from the optic chiasma or optic nerve.

### Statistical analysis

Descriptive parameters, including the mean, median, counts, and proportions, were calculated with Python 3.7. The mean was used to describe the continuous variables that fit a normal distribution, and the median and quartile are used to describe the continuous variables that did not fit a normal distribution. The Chi-square test was used to test the difference for categorized variables. Kruskal-Wallis test and Mann-Whitney test were used to detect differences for continuous variables among multi-groups and two groups respectively. Kaplan–Meier analysis was used to compute the survival rate.

## Results

**Figure 1:**
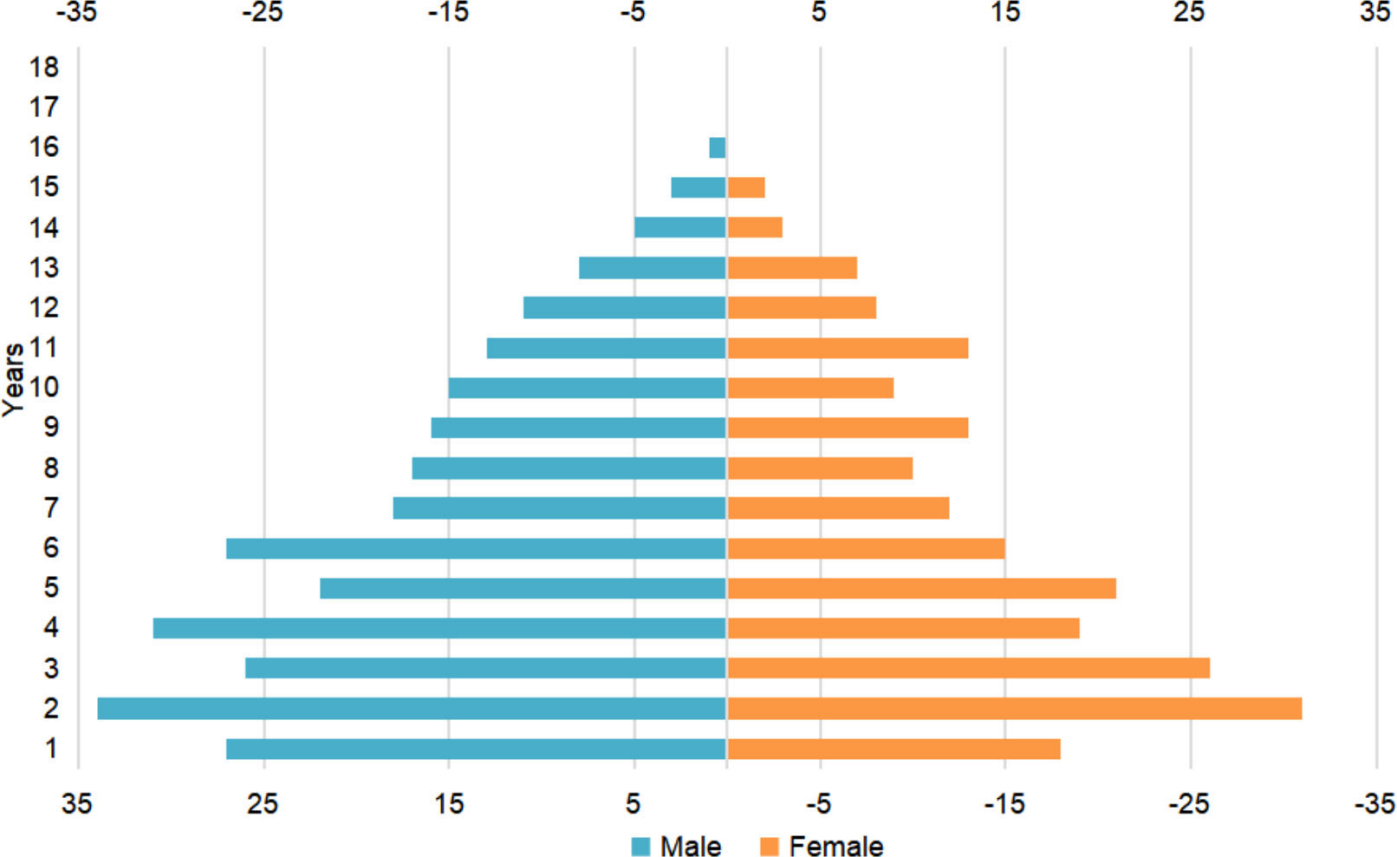
Age distribution for males and females in Beijing Children’s Hospital from 2015 to 2020.

From 2015 to 2020, 484 individuals aged between 0-16 years were diagnosed with brain tumors and underwent neurosurgery in our center. The median age of this cohort was 4.62 [2.19, 8.17] years, with males making up 57.0% of the cohort. The median ages at diagnosis of the male and female groups were 4.79 [2.26, 8.12] and 4.55 [2.14, 8.19] years respectively, not significantly different (p>0.05). Benign tumors accounted for 53.1% of all brain tumors; the median age for these patients was 4.07 [1.64, 7.13] years and the male-to-female ratio was 1.09:1. Distinct from benign brain tumors, patients with malignant brain tumors (46.9%) had a median age of 5.36 [2.78, 8.84] years and a male-to-female ratio of 1.62:1. Mann-Whitney U test and chi square test found significant difference in age (p=0.008) and sex (p=0.029, chi square =4.743) among the malignant and benign groups. **Figure 1** showed the overall age distribution among male and female. Overall, the average length of hospital stay was 19.18 days, and the average medical expenditure was 10903.43 $.

### Clinical presentations

Overall, the most frequent initial symptom was nausea and vomiting (24.0%), followed by headache (23.4%), motor impairment (12.6%), and epilepsy (10.5%). Other symptoms, such as visual impairment, behavior change, growth or endocrinal abnormity, accounted for less than 10% of the symptoms. For posterior fossa tumors, the most frequent symptom was headache (32.4%), followed by nausea and vomiting (32.4%), and motor impairment (13.5%). Among individuals with sellar tumors, visual impairment (20.9%) was the most common symptom, followed by headache (15.5%), motor impairment (14.7%), nausea and vomiting (14.0%), and tumor growth or endocrinal abnormity (14.0%) (see **Supplementary Table 1**).

Patients with temporal lobe tumors experienced the longest median duration of 18 [4, 51] weeks while meningioma had the shortest duration of 2 weeks. The median duration of symptoms of patients with sellar tumors was 13 [4, 52] weeks and 4 [3, 13] weeks for patients with cerebellum or fourth ventricle tumors. Details were shown in **Supplementary Table 2**. According to the records, approximately 35.2% (167/475, 9 records did not have a clear description of the medical history and were excluded) of the patients were misdiagnosed in their first visit to the hospital. The top three pathology types most likely to be misdiagnosed were pineal tumors (misdiagnosis rate[cases misdiagnosed of pineal tumors/all cases of pineal tumors]: 60.0%), glioneuronal and neuronal tumors (40.5%), and embryonal tumors (40.0%). The top three tumor location most likely to be misdiagnosed were temporal lobe (48.6%), ventricles (42.9%), and cerebellum or fourth ventricular (37.2%).

### Distribution of tumor pathology

**Figure 2:**
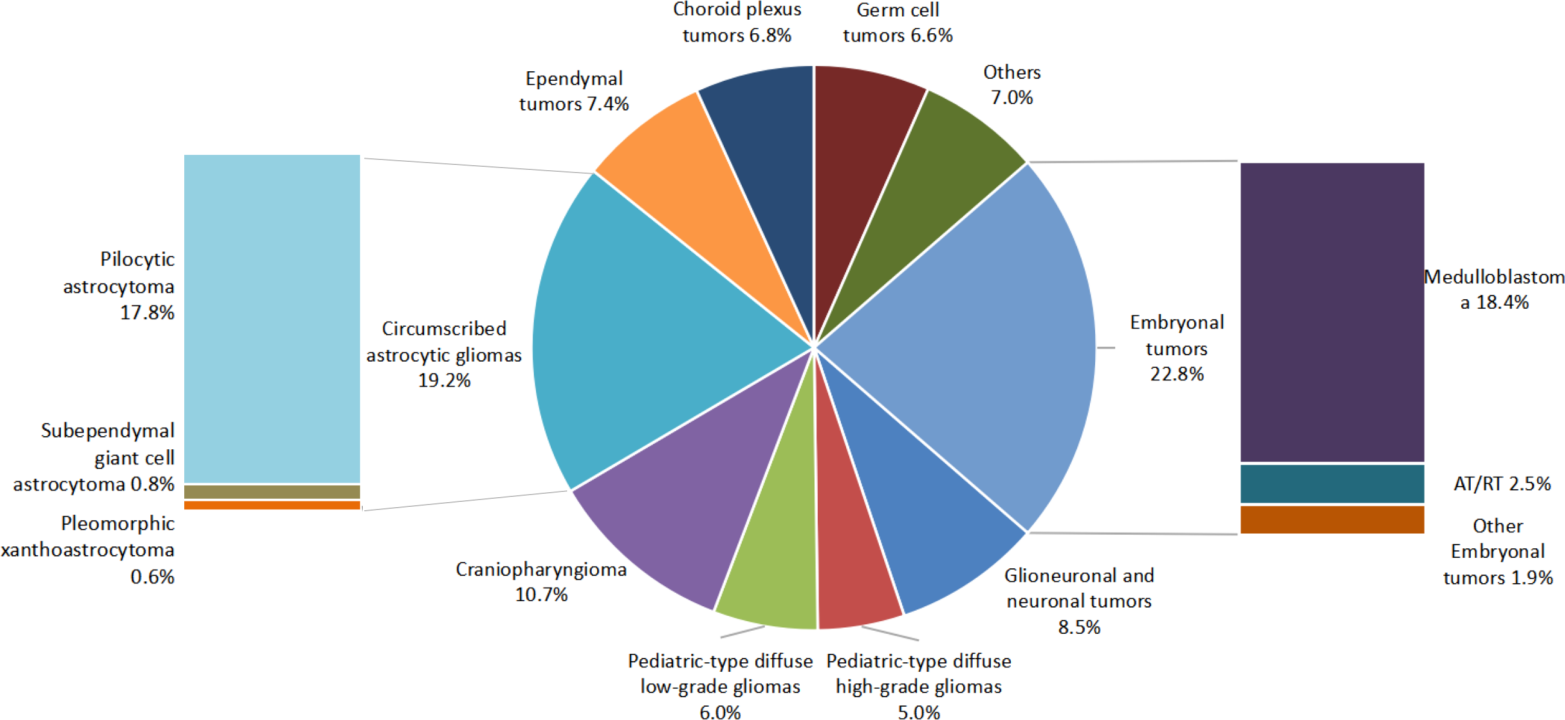
Pathology distribution of brain tumors in Beijing Children’s Hospital from 2015 to 2020.

Based on the 2021 CNS WHO classification, the most common tumor type was ET (22.5%), followed by circumscribed astrocytic gliomas (20.0%), pediatric-type diffuse gliomas (11.0%) and craniopharyngioma (CP, 10.7%), see **Figure 2**. Among benign tumors, the three most common types were circumscribed astrocytic gliomas (37.5%), CP (21.7%), and glioneuronal and neuronal tumors (14.6%). ET (44.8%) were the most common type in the malignant brain tumor group, followed by pediatric-type diffuse gliomas (22.0%) and ependymal tumors (14.5%). Among ET, the most common pathology was medulloblastoma (MB), accounting for 82.4% of the tumors.

### Distribution of tumor sites

**Figure 3:**
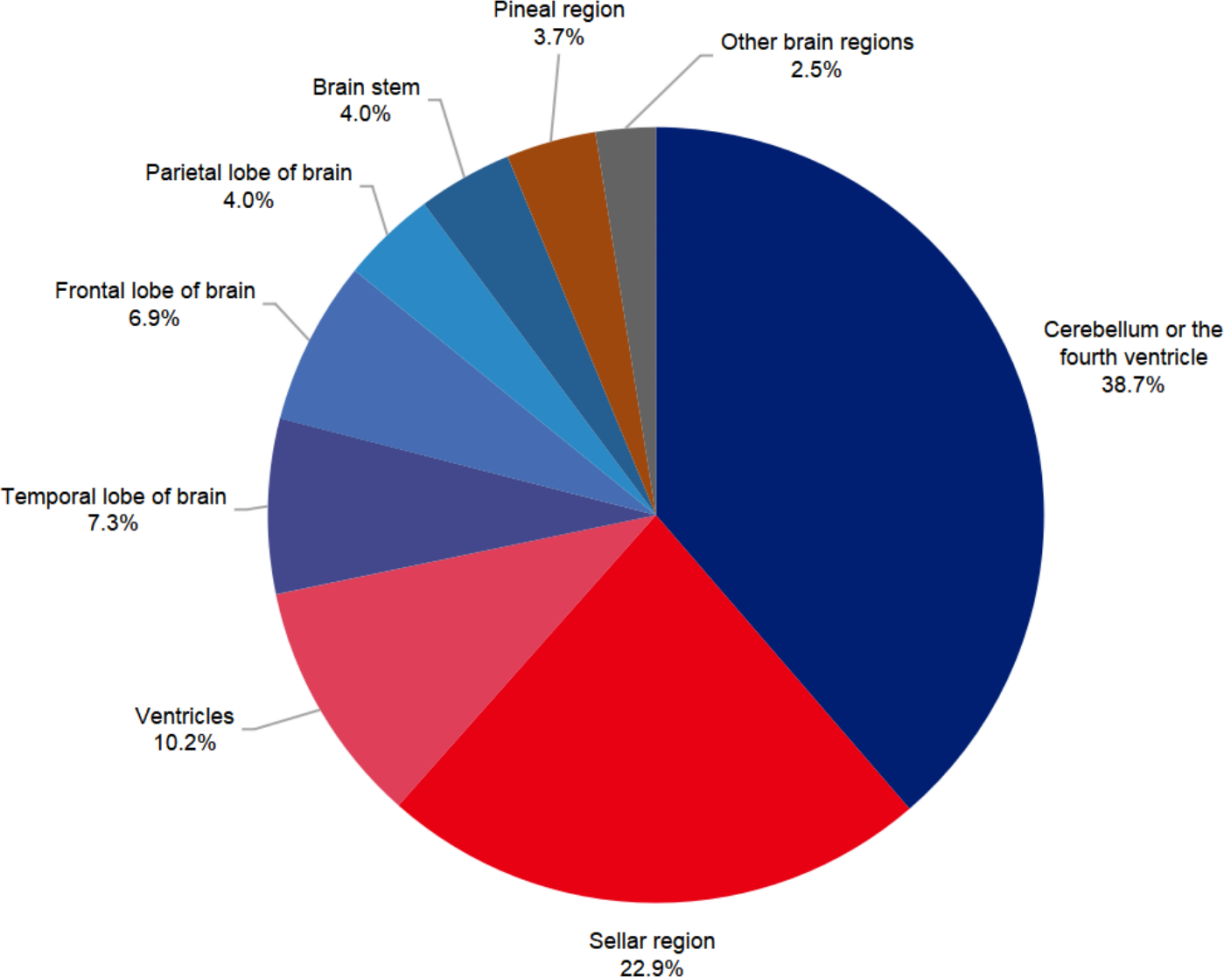
Sites distribution of pediatric brain tumors in Beijing Children’s Hospital from 2015 to 2020.

Overall, cerebellum or the fourth ventricle was the most common site of brain tumors (38.7%), followed by the sellar region (22.9%). Details were shown in **Figure 3**. However, tumors in supratentorial space in sum (57.4%) still comprised more than half of all brain tumors. Benign and malignant tumors had significantly different patterns of site distribution (p<0.001). In the benign tumor group, the most common site was the sellar region (40.0%), followed by the cerebellum or the fourth ventricle (24.6%) and ventricles (17.1%). Regarding the malignant brain tumors group, the cerebellum or the fourth ventricle (52.7%) were the most common sites, with other sites sharing an even proportion.

**Figure 4:**
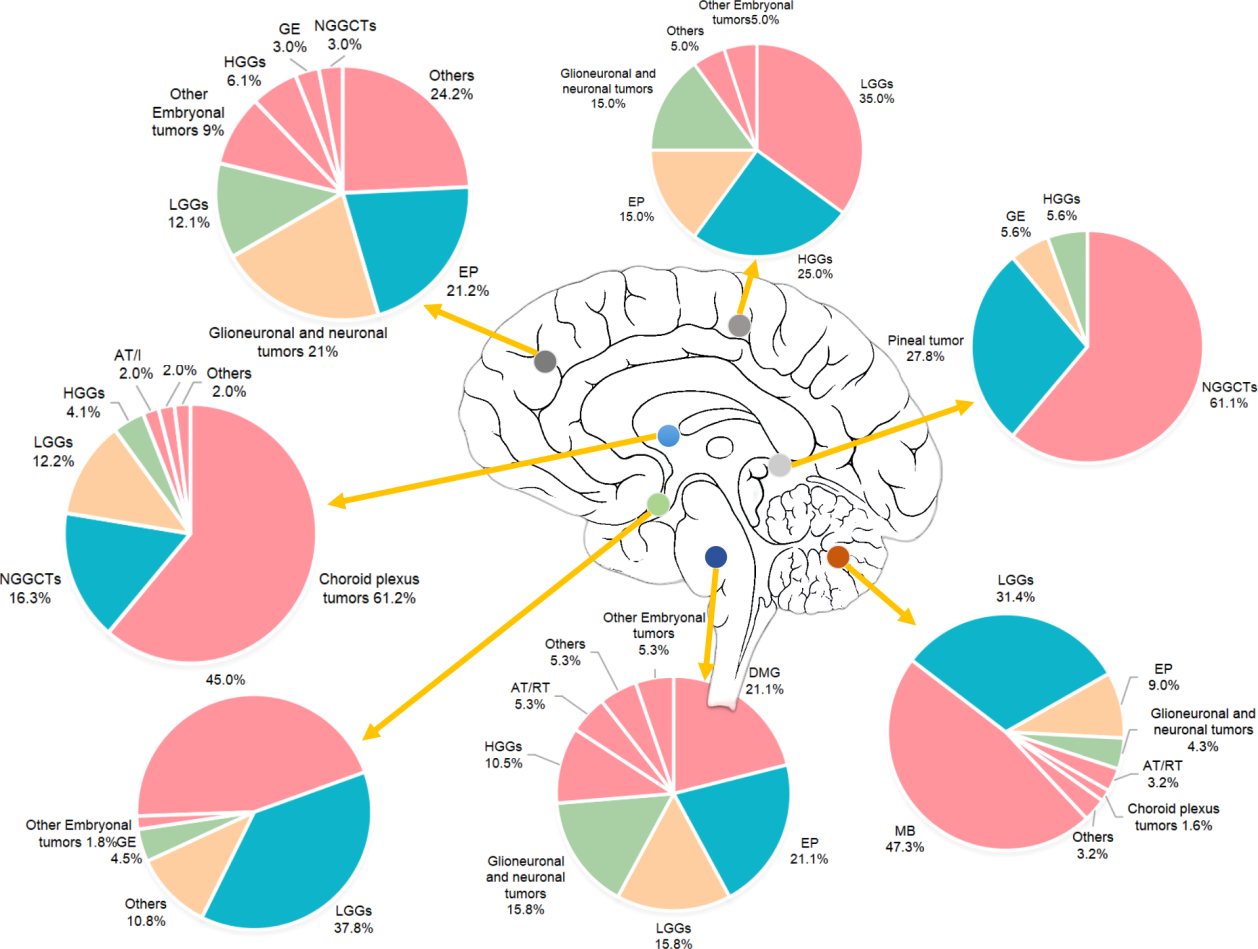
Pathology distributions in different brain regions.

In different regions of brain, the pathology distribution was various (see **Figure 4**). In the cerebellum or fourth ventricle, MB (43.7%) were the most common tumor type, accounting for near half of the total tumors. The second and third most common tumor types in this hospital were low grade gliomas (31.4%) and ependymal tumors (9.0%). In the sellar region, the three most common pathologies were CP (45.0%), low grade gliomas (37.2%).

### Sex and age distribution

Across all brain tumors, the proportion of males (57.17%) was slightly greater than that of females (42.83%). The sex distribution varied greatly with different tumor pathologies, despite no significant difference was identified among different pathological types (p>0.05). Males (52.08%) and females (47.92%) shared a similar proportion of benign tumors, while malignant tumors were more common in males (62.24%) than females (37.76%). Specifically, there was no obvious sex bias for circumscribed astrocytic gliomas (Male: Female, 47.4% vs. 52.6%), Pediatric-type diffuse gliomas (50.9% vs. 49.1%), glioneuronal and neuronal tumors (51.4% vs. 48.6%) or CP (51.9% vs. 48.1%). However, a higher proportion of males than females were diagnosed with ET (61.1% vs. 38.9%), ependymal tumors (68.6% vs. 31.4%), pineal tumors (100.0% vs. 0%), GCTs (78.1% vs 21.9%), and choroid plexus tumors (63.6% vs. 36.4%) than females. Malignant tumors have slightly but significantly higher rate of male proportion compared with female (61.9% vs. 52.1%, p=0.029).

The median age varied significantly with different histological types of brain tumors (p<0.001, see **Table 1**). Patients with ependymal tumors (4.10[2.14, 7.60]), ET (5.93[2.96,8.87]), GCTs (9.30[4.15, 10.99]), CP (7.74[3.53,8.21]), meningiomas (11.98[7.85, 13.19]), and metastatic tumors (5.54[4.96, 6.88]) had a median age older than 3 years, while patients with pineal tumors (1.52[1.49, 1.77]) and choroid plexus tumors (0.73[0.44, 1.51]) were generally younger than 3 years of age. In ET, an age difference was noted. The median age of MB patients was 6.93[4.12, 9.75] years, while that of AT/RT patients was 2.30[1.34, 2.74] years. In all patients, malignant tumors had a older age than begin tumors (5.36[2.79, 8.84] vs. 4.08[1.64, 7.13], p<0.001).

**Table 1:** Sex, age and sites distribution of brain tumors.

	Males, n (%)	Median age (years)	Most frequent site
Choroid plexus tumors (N=33)	12 (63.6)	0.73 (0.44, 1.51)	Ventricles
Melanocytic tumors (N=1)	1 (100.0)	1.69 (1.69, 1.69)	Frontal lobe of brain
Tumors of pineal region (N=5)	5 (100.0)	1.52 (1.49, 1.78)	Pineal region
Mesenchymal, non-meningiothelial tumors (N=4)	2 (50.0)	2.92 (2.35, 4.59)	Other brain regions
Neuronal and mixed neuronal-glial tumors (N=37)	19 (51.4)	3.85 (1.31, 6.49)	Temporal lobe of brain
Other astrocytic tumors (N=97)	46 (47.4)	4.10 (1.80, 7.09)	Cerebellum or the fourth ventricle
Ependymal tumors (N=36)	24 (68.6)	4.26 (2.17, 7.62)	Cerebellum or the fourth ventricle
Diffuse astrocytic and oligodendroglial tumors (N=53)	27 (50.9)	5.30 (2.89, 7.69)	Cerebellum or the fourth ventricle
Others (N=13)	7 (53.8)	4.38 (3.90, 8.18)	Sellar region
Craniopharyngioma (N=52)	27 (51.9)	5.74 (3.53, 8.21)	
Embryonal tumors (N=110)	66 (61.1)	6.08 (3.14, 8.98)	Cerebellum or the fourth ventricle
Metastatic tumors (N=7)	3 (42.9)	5.55 (4.96, 6.88)	Frontal lobe of brain
Tumors of Cranial and paraspinal nerves (N=1)	1 (100.0)	7.07 (7.07, 7.07)	Cerebellum or the fourth ventricle
Germ cell tumors (N=32)	25 (78.1)	9.30 (4.15, 10.99)	Pineal region
Meningiomas (N=3)	2 (66.7)	11.98 (7.85, 13.19)	Meninges
Benign tumors (N=240)	125 (52.1)	4.08 (1.64, 7.13)	Sellar region
Malignant tumors (N=244)	151 (61.9)	5.36 (2.79, 8.84)	Cerebellum or the fourth ventricle
All tumors (N=484)	276 (57.0)	4.62 [2.19, 8.17]	Cerebellum or the fourth ventricle

### Treatment and survival

All the patients received tumoral resection. The adjuvant therapy varies among different types of tumors, while a primary principle was followed: Generally, a gross total or extensive resection and regular outpatient surveillance were a priority for most of the brain tumors except for optic pathway gliomas and diffuse intrinsic pontine gliomas. Chemotherapy and radiotherapy were administered for all malignant tumors and optic pathway gliomas and diffuse intrinsic pontine gliomas under radiologists’ and chemotherapists’ suggestions. For metastatic ET, the gross total resection was also the priority followed by chemotherapy and/or radiotherapy, while the biopsy was not recommended. In addition, children under 3 years old was not recommended for radiotherapy as the serious cognitive impairment effect. And adjuvant therapy was not recommended for CP as gross total resection was encouraged in our center in the past clinical practice and almost all the patients reached gross total resection. Recent studies have shown that subtotal resection followed by proton therapy can reach a similar survival curve but with less morbidity (6–8). It is changing the surgical concept of CP. However, not all the patients followed doctors’ advice after surgery. **Table 2** summarized the treatment profile of these patients.

**Table 2:** Treatment options and survival rate of main tumor groups.

Pathology	Radiotherapy	Chemotherapy	Median survival time (Month)	1-year survival rate (%)	5-year survival rate (%)
All tumors (N=437), n (%)	137 (34.1)	146 (40.8)	–	89.0	77.5
Malignant tumors (N=211), n (%)	137 (64.9)	146 (69.2)	76.0	81.0	64.6
Benign tumors (N=216), n (%)	12 (5.6)	33 (15.3)	–	97.5	91.0
MB (N=80), n (%)	65 (81.3)	68 (85.0)	76.0	86.7	70.5
AT/RT (N=10), n (%)	5 (50.0)	7 (70.0)	3.0	0	0
Other ET (N=8), n (%)	4 (50.0)	5 (62.5)	14.0	50.0	37.5
EP (N=34), n (%)	26 (76.5)	23 (67.6)	–	97.1	78.2
DMG (N=6), n (%)	3 (50.0)	3 (50.0)	5.0	13.1	0
LGG (N=105), n (%)	11 (10.5)	34 (32.4)	–	94.3	89.5
HGG (N=13), n (%)	8 (61.5)	10 (76.9)	27.0	62.2	17.9
GE (N=6), n (%)	4 (66.7)	4 (66.7)	–	91.7	80.0
NGGCTS (N=21), n (%)	11 (52.4)	12 (57.1)	–	89.7	89.7
CP (N=52), n (%)	0 (0)	0 (0)	–	100.0	87.7

Not all tumor types are listed in this table. Patients lost for follow up were not included in this table.

MB, medulloblastoma; AT/RT, Atypical Teratoid Rhabdoid Tumors; ET, embryonal tumors; EP, ependymoma; DMG, diffuse midline glioma; LGGs, low grade gliomas; HGGs, High grade gliomas; GE, Germinoma; NGGCTS, Nongerminomatous Germ Cell Tumors; CP, craniopharyngioma.

All the patients were followed up for 1 to 82 months. There were 54 patients lost during the follow-up. The overall 5-year survival rate was 77.4% in the 430 patients with brain tumors, with a median survival time of 81.0 months. Patients with malignant and benign tumors had 5-year survival rates of 64.6% and 91.0%, respectively. The median survival time of patients with malignant tumors was 76.0 months. **Table 2** shows survival rate of several main tumor groups. Given that more than half of the patients remained alive, it was not feasible to calculate the median survival time of patients for some tumor groups.

## Discussion

Central nervous system tumors account for a quarter of all childhood cancers and are the most common solid tumors, of which brain tumors account for the majority (9–11). In the United States, brain tumors make up more than 1% of newly diagnosed cancer cases (12). Despite improved treatment in recent years, brain tumors are still the leading cause of cancer-related death among children (13). Brain tumors are heterogeneous and vary by race, sex, age and so on (14–16). Epidemiological studies in China are scarce and are based on the dated CNS WHO classification (4). Hence, it is meaningful to summarize and validate the epidemiological information again. In addition, this study collected and described other important information, such as medical expenditures, manifestations, and prognoses.

### Presentations

The most frequent initial symptoms were nausea, vomiting and headache. Symptoms of motor impairment were present in only 12.59% of all children with brain tumors. The results are consistent with those of a previous meta-analysis study (17). Manifestations are associated with brain tumor locations. Visual impairment is frequently seen in patients with sellar tumors, while nausea and vomiting and headache are more common in patients with cerebellum and fourth ventricle tumors. Misdiagnosis and delay in diagnosis might occur because these common symptoms are not specific to CNS tumors; our data showed that the rate of misdiagnosis reached 32.5%. Supratentorial tumors are more insidious than infratentorial tumors. The general median duration of symptoms across all children brain tumors was 4 weeks, while children with tumors of the temporal lobe experienced the longest duration, with a median of 18 weeks. The general median symptom duration of children with posterior fossa tumors was 4 weeks. This result implies that the diagnostic capability in China has reached that of the international level (18). However, the longest symptom duration of children with posterior fossa tumors was more than 10 years. Increasing awareness of the varied and complex symptomatology that often occurs with CNS tumors in China is necessary and could help reduce misdiagnosis and achieve early diagnosis. Prompt cranial imaging examinations for children with unknown headache and nausea is necessary.

### Predilection age

The number of brain tumors decreased with advancing age, which is consistent with the CBTRUS 2015 report (apart from tumors of the pituitary gland) (3). The median age of the total group was 4.62 years, with a median age of 5.36 years for the malignant tumor group, which was slightly higher than that of the benign group. This trend is consistent with a previous report (16). However, the recent CBTRUS report indicates that malignant tumors, compared with nonmalignant tumors, tend to affect younger children (1). This might be due to the sampling bias for tumor histology. Benign pituitary gland tumors tend to affect adolescents, which causes an increase in the median age of the benign tumors group; participants in this age group were not sufficiently enrolled in our study. Our data show that CP, ependymal tumors, ET, metastatic tumors, GCTs, meningiomas and cranial and paraspinal nerve tumors tend to affect older children, while choroid plexus tumors, melanocytic tumors, pineal tumors, glioneuronal and neuronal tumors, and circumscribed astrocytic gliomas tend to affect infants and toddlers. This is in accordance with previous CBTRUS reports (19).

### Sex

The sex distribution across all brain tumors is almost balanced. However, in the subgroup analysis, a significant greater proportion of malignant tumors than benign tumors were present in males. This is in accordance with a previous report that malignant tumors occur much more frequently in males (14). We found that sex differences varied by histology. GCTs, ET, ependymal tumors, and choroid plexus tumors were observed in more males than females, while little sex bias was noted for gliomas, and glioneuronal and neuronal tumors and CP. These results are similar to the data of the CBTRUS report (1). These results might also inspire researchers to study the harmonic effect in the pathogenesis of brain tumors.

### Location

Similar to previous studies, supratentorial tumors were more slightly common (57.4%) than infratentorial tumors (42.6%). Specifically, the three most common sites were the cerebellum or fourth ventricle, the sellar region and ventricles. This was different from the CBTRUS report (1), which showed that the three most common sites are the sellar region, cerebellum and other brain regions. Furthermore, the proportion of tumors in the cerebellum in our center was approximately two times that in the US. This might be due to that we classified tumors in the cerebellum and fourth ventricle into one group, but the CBTRUS report classifies tumors in the fourth ventricle and cerebellar tumors as two groups. To have a firm conclusion, a national wide data source is necessary in future studies. Given the difficulty in differentiating tumors of the cerebellum or the fourth ventricle in magnetic resonance imaging (MRI), we believe it is more applicable to categorize these two sites into one group. When looking at the nonmalignant and malignant groups, the site distribution pattern was different. The most common site in the malignant tumors group was the cerebellum or fourth ventricle, while the sellar region was the most common site in the benign tumors group. The pathology distribution patterns in posterior fossa, sellar region and ventricles were in accordance with previous studies (20). With these figures, our data might help clinicians have a better understanding of the differential diagnosis of pediatric brain tumors.

### Pathology

Different from the existing studies, this study was based on the WHO 2021 classification. Tumors accounting for less than 5% of all brain tumors were assigned to the “others group”. Above all, the most common pathology type was ET, accounting for 22.5% of all brain tumors, followed by other astrocytic tumors and diffuse astrocytic and oligodendroglial tumors. This is consistent with a previous study of the Chinese population (4). However, the CBTRUS reported that pilocytic astrocytoma is the most common pathology type (1, 3, 14, 19). This still need to be validated by more studies in future. A potential cause for this might be different approaches of classification. According to the WHO 2021, we included all ET into one group, leading to an increase in the proportion of ET while CBTRUS did not classify in this way. Distinct from a previous study in China (4), we showed that MB were more frequent than CP, and ependymal tumors were more common than GCTs, which is consistent with previous reports (19, 21, 22). We speculate that there might be sample bias in the previous Chinese report because it was not a children’s hospital, the younger patients might prefer to attend a children hospital rather than a general hospital. GCTs only account for 6.7% of whole-brain tumors, which is similar to the results of an investigation in China but different from other reports in Japan, Taiwan (China) and far eastern countries with an incidence of brain tumors of 10–14%. This difference might be explained by the fact that patients with germinoma (GE) often undergo nonsurgical treatments, and the actual number of GCTs might have been significantly underestimated in this study. The WHO 2021 address the importance of the molecular scope of brain tumors and classify these tumors into different molecular groups. The medulloblastoma has been divided into four different molecular types, and they are closely related to the prognosis and treatment regimen. The current molecular classification has been used to stratify the treatment intensity. KIAA1549–BRAF fusion is found in 80% of all LGGs whereas only about 10%-20% LGGs possess BRAF-V600E mutation (23). Studies have indicated that BRAF inhibitors could lead to a partial response in patients with BRAF aberrant pilocytic astrocytoma (24). The molecular classification not only provide specific treatment, but also help stratify the patients into different risk categories (25, 26). However, it is a pity that, due to our data being retrospective collected, the molecular diagnosis was absent. Despite of this, our current clinical work flow has introduced the molecular diagnosis. We hope to report our data in future research. Besides, due to the lack of a national wide brain tumor registration system for children and few epidemiological reports, we have to explain these differences based on experience. A registration system for brain tumors is urgent and necessary in China.

### Survival

It is known that the survival of patients with brain tumors varies by histology, age at diagnosis, tumor location, and so on. Our data showed that the 5-year overall survival (OS) rate of patients with benign tumors was 90.0%, consistent with previous studies. In the United States, 96% of the children aged 0–19 years with nonmalignant tumors survived 10 years after diagnosis (27). Tore Stokland (28) reported that the 5-year OS rate of children with LGGs was up to 96.4%. However, the outcomes could vary with the extent of resection. If complete surgical resection is performed, the 10-year progression-free survival (PFS) rate exceeds 85% but it drops below 50% if there is radiologically visible residual tumor (29). Sahaja Acharya (30) reported that the 10-year OS of children with LGGs reached 76.4% (high risk) ~ 95.6% (low risk). Alvaro Lassaletta (31) reported that the 10-year PFS was 27% and 60.2% for the BRAF VE600 mutation and wild-type cohorts, respectively. The OS rate of patients with CP ranges from 83% to 96% at 5 years (32) and from 65% to 100% at 10 years (33–35) and is, on average, 62% at 20 years. At present, whether age at diagnosis of CP, sex, and pathological subtype are prognostic factors for survival remains controversial (8).

The 5-year OS rate of children with malignant tumors was 64.6%, similar to previous reports (75.4%) (1). The 10-year survival rate for children aged 0–19 years diagnosed with malignant brain and other CNS tumors was estimated at 72%, with the lowest rate (17%) attributed to glioblastoma (16). Other studies reported that less than 5.5% of glioblastoma patients survived more than 5 years (36, 37). Another malignant tumor, diffuse intrinsic pontine gliomas, generally has an OS rate of less than 1 year (38). Children with atypical teratoid/rhabdoid tumors were reported to have a four-year OS rate of 43% (39), and another study reported a 6-year OS rate of 35% (40). High-dose chemotherapy and radiation therapy were associated with better survival, while tumor metastasis, intrathecal chemotherapy and the extent of resection did not significantly affect survival (41). Overall, the prognoses of malignant tumors remain unsatisfactory, and more resources need to be introduced in this field. Due to the limitation of the sample size, we did not calculate the survival rate of the specific tumor types. We hope to perform this analysis in future studies.

To date, little information of the epidemiological characteristics of Chinese people is known. As a national center for children’s health, we summarized our data and hope that our experiences will provide more information about pediatric brain tumors in China. Moreover, we acknowledge that due to the lack of a nationwide registration system for brain tumors, some inevitable bias might exist. Apart from this, children aged 15-18 years in China prefer to attend general hospitals rather than children’s hospitals. Hence, the number of adolescent patients was relatively small in this study.

## Conclusion

Overall, the epidemiological characteristics of brain tumors in our center presented a similar pattern to those in previous reports. Some difference was noted and are needed to be confirmed by more epidemiological studies in future.The ratio of benign to malignant tumors approached 1:1.03. Males were more vulnerable to malignant tumors. The site distribution patterns of benign and malignant brain tumors were significantly different. These demographic characteristics provide us with further understanding of pediatric brain tumors, such as sex predispositions and predilection age of onset. Our data might be able to reflect the actual situation of pediatric brain tumors in China.

## Data availability statement

The raw data supporting the conclusions of this article will be made available by the authors, without undue reservation.

## Ethics statement

The studies involving human participants were reviewed and approved by Ethic committee board of Beijing Children’s Hospital. Written informed consent for participation was not provided by the participants’ legal guardians/next of kin because: This is a retrospective study and no identical participant information was included in our manuscript and our institutional ethic committee waived the request for written consent.

## Author contributions

MG, WY, and YC contributed to the study conception and design. Material preparation, data collection and patient follow up were performed by JC, PY, ZY, YL, ML, HS, YJ, XP, and data analysis was performed by WY, KZ and WM. Pathology reconfirmation was performed by NZ. The first draft of the manuscript was written by WY, and all authors commented on previous versions of the manuscript. All authors contributed to the article and approved the submitted version.
