# Supplementary material for: Epidemiological characteristics, clinical presentations, and prognoses of pediatric brain tumors: Experiences of national center for children’s health
Source: Front Oncol. 2023 Jan 27;13:1067858. doi: 10.3389/fonc.2023.1067858 (PMC9915562; doi:10.3389/fonc.2023.1067858)
Supplement: Supplementary file 1 [file Table_1.docx]

**Supplementary table 1 Summaries of Initial symptoms**

| **Initial symptoms** | **Overall (n, %)** | | **Cerebellum or fourth ventricles (n, %)** | | **Sellar region (n, %)** | | **Ventricles (n, %)** | |
| --- | --- | --- | --- | --- | --- | --- | --- | --- |
| Nausea and vomiting | 137 | 24.0% | 72 | 32.4% | 18 | 14.0% | 14 | 25.9% |
| Headache | 134 | 23.4% | 81 | 36.5% | 20 | 15.5% | 6 | 11.1% |
| Motor impairment | 72 | 12.6% | 30 | 13.5% | 19 | 14.7% | 6 | 11.1% |
| Epilepsy | 60 | 10.5% | 6 | 2.7% | 6 | 4.7% | 3 | 5.6% |
| Visual impairment | 54 | 9.4% | 12 | 5.4% | 27 | 20.9% | 6 | 11.1% |
| By accident | 33 | 5.8% | 7 | 3.2% | 7 | 5.4% | 7 | 13.0% |
| Abnormal behaviors | 29 | 5.1% | 5 | 2.3% | 8 | 6.2% | 5 | 9.3% |
| Growth and [endocrine](javascript:;) [dyscrasia](javascript:;) | 25 | 4.4% | 2 | 0.9% | 18 | 14.0% | 2 | 3.7% |
| Cranial nerve impairment | 9 | 1.6% | 2 | 0.9% | 2 | 1.6% | - | - |
| Abnormal head appearance | 9 | 1.6% | 1 | 0.5% | 1 | 0.8% | 4 | 7.4% |
| Head tilt | 6 | 1.1% | 4 | 1.8% | - | - | - | - |
| Abnormal temperature | 3 | 0.5% | - | - | 3 | 2.3% |  |  |
| [Pat the head](javascript:;) | 1 | 0.2% | - | - | - | - | 1 | 1.9% |
