# Supplementary material for: Epidemiological characteristics, clinical presentations, and prognoses of pediatric brain tumors: Experiences of national center for children’s health
Source: Front Oncol. 2023 Jan 27;13:1067858. doi: 10.3389/fonc.2023.1067858 (PMC9915562; doi:10.3389/fonc.2023.1067858)
Supplement: Supplementary file 2 [file Table_2.docx]

**Supplementary table 2 Duration of symptoms**

|  | **Duration of symptoms (weeks)** | | | | |
| --- | --- | --- | --- | --- | --- |
| **Tumor Location** | **min** | **25%** | **50%** | **75%** | **max** |
| Temporal lobe of brain（N=35） | 0 | 4 | 18 | 51 | 365 |
| Other brain regions (N=9) | 3 | 4 | 14 | 35 | 365 |
| Occipital lobe of brain (N=1) | 13 | 13 | 13 | 13 | 13 |
| Sellar region (N=104) | 0 | 4 | 13 | 52 | 309 |
| Parietal lobe of brain (N=19) | 0 | 1.5 | 9 | 11.5 | 77 |
| Pineal region (N=16) | 0 | 2 | 5 | 10 | 51 |
| Brain stem (N=19) | 0 | 1.5 | 4 | 5 | 77 |
| Cerebellum or the fourth ventricle (N=186) | 0 | 3 | 4 | 13 | 617 |
| Frontal lobe of brain (N=33) | 0 | 3 | 4 | 10 | 104 |
| Ventricles (N=51) | 0 | 2 | 3 | 9 | 261 |
| Meninges (N=2) | 0 | 1 | 2 | 3 | 4 |

Note: Sellar region includes optic gliomas originated from chiasm；4th ventricles was not included in ventricles.
